# Teat sealant cannula insertion length is not associated with postcalving mastitis risk in cattle

**DOI:** 10.1002/vetr.5226

**Published:** 2025-03-19

**Authors:** Cherrill Bedford, Philippa Jane Mahen, Kath Aplin, George Oikonomou

**Affiliations:** ^1^ Department of Livestock and One Health Institute of Infection Veterinary and Ecological Sciences University of Liverpool Neston UK; ^2^ Boehringer Ingelheim Animal Health Penrith UK

**Keywords:** dairy cattle, mastitis, somatic cell counts

## Abstract

**Background:**

Internal teat sealants are commonly used at drying off, with or without intramammary antibiotics, to reduce the risk of mastitis. Both full and partial cannula options are available on most teat sealant and antibiotic tubes, but little evidence exists to support the selection of one option over the other.

**Methods:**

A total of 287 Holstein cows from three UK farms were enrolled in the study. Cows were randomly allocated to receive either full or partial insertion of internal teat sealant at drying off (plus the same insertion type of intramammary antibiotic if required by farm protocols). Somatic cell count and clinical mastitis data were collected, along with lactation number and calving season, and analysed using multivariable regression modelling.

**Results:**

Insertion type was not associated with high postcalving somatic cell counts (SCCs; >200k cells/mL), new infection rates (low to high SCC change across the dry period), cure rates (high to low SCC change across the dry period) or clinical mastitis cases.

**Limitations:**

Only pedigree Holsteins were included; results in other breeds could differ due to teat size.

**Conclusion:**

Insertion type was not associated with high SCC or increased postcalving mastitis cases.

## INTRODUCTION

Mastitis is a major cost to UK dairy herds and is one of the main factors affecting whether a cow will be culled involuntarily (i.e. forced culling).^1^, ^2^ A study on 97 UK dairy farms estimated the number of clinical cases of mastitis at 47 per 100 cows per year from farm records; however, milk sampling from the same study suggested that the number of clinical cases was as high as 71 cases per 100 cows per year.^3^ Researchers in the United States estimated that the average cost of a case of clinical mastitis was $444 (∼£342) in direct and indirect costs.^4^

The dry period is a high‐risk period for udder health, with more than 60% of new cases of mastitis originating during the dry period.^5^ Antibiotics (ABs) have been used at drying off since the 1950s, but due to increasingly successful control of contagious mastitis and the emerging issue of antimicrobial resistance, there is pressure to reduce the use of ABs at drying off.^6^ Teat sealant tubes are designed to be administered at drying off, with or without the use of AB tubes, and form a physical barrier to mastitis‐causing pathogens. A meta‐analysis of 12 studies on the effects of teat sealants on intramammary infections found that ‘internal teat sealants (ITSs), alone or in the presence of antibiotic dry cow therapy (ADCT), reduced the risk of acquiring new intramammary infections after calving by 25%’.^7^

ITS and intramammary AB application tubes often have insertion cannulas that can be used either as short or long cannulas. In a cohort study in Thailand, full cannula insertion of ADCT has been associated with a higher risk of postcalving mastitis.^8^

A study in the 1980s, involving postmortem histological exploration of the differences between using full and partial cannula insertion in Jersey cows, found a significantly higher new infection rate for full cannula insertion of ADCT compared with partial insertion (PI). In addition, histological samples showed a partial loss of the keratin lining of the teat canal when fully inserting the cannula.^9^

However, there is a lack of published work directly comparing the effects of using full or partial cannula insertion for the administration of ITS. We aimed to test the hypothesis that the fully inserted long cannula could increase the risk of introducing new infections into the udder, leading to higher somatic cell counts (SCCs) postcalving and a greater period prevalence of mastitis in the first 30 days postcalving by comparing the full insertion (FI) and PI of the cannulas in a randomised controlled trial.

## MATERIALS AND METHODS

The study design was created with the aim of recruiting 250 cows per group, which would allow the detection of a change in SCC of 50,000 cells/mL (confidence level: 0.95; power: 0.80).

A convenience sample of three pedigree Holstein dairy farms in the UK (A, B and C) was selected to take part in the study over a period of 6 months (from March to August 2019). Farm A milked approximately 180 cows three times a day with a 305‐day average yield of 11,000 L. Milking cows were cubicle housed all year and bedded on mattresses with sawdust, and dry cows were housed in straw yards. Farm B milked approximately 340 cows twice a day with a 305‐day average yield of 9000 L. High yielding cows were cubicle housed on mattresses with sand, and low yielding cows were grazed; dry cows were also grazed for the first part of their dry period and then moved to straw yards. Farm C milked approximately 280 cows twice a day with a 305‐day average yield of 8800 L. Milking cows were grazed in summer and cubicle housed in the winter. During the summer, dry cows were grazed for the first part of their dry period and then moved to straw yards. In the winter, the whole dry period was spent in straw yards. All three farms had all‐year‐round calving patterns; however, farm B had an increased number of first lactation animals calving in late spring/early summer.

All three farms milked in herringbone parlours with automatic cluster removal systems. Farm A used teat wipes to clean lightly soiled teats and pre‐dip on heavily soiled teats; after milking, teats were sprayed manually with a lactic acid‐based product. Farm B used peracetic acid and teat conditioner as a pre‐brush; after milking, teats were sprayed with an iodine solution. Farm C sprayed with an iodine spray after milking.

**FIGURE 1:**
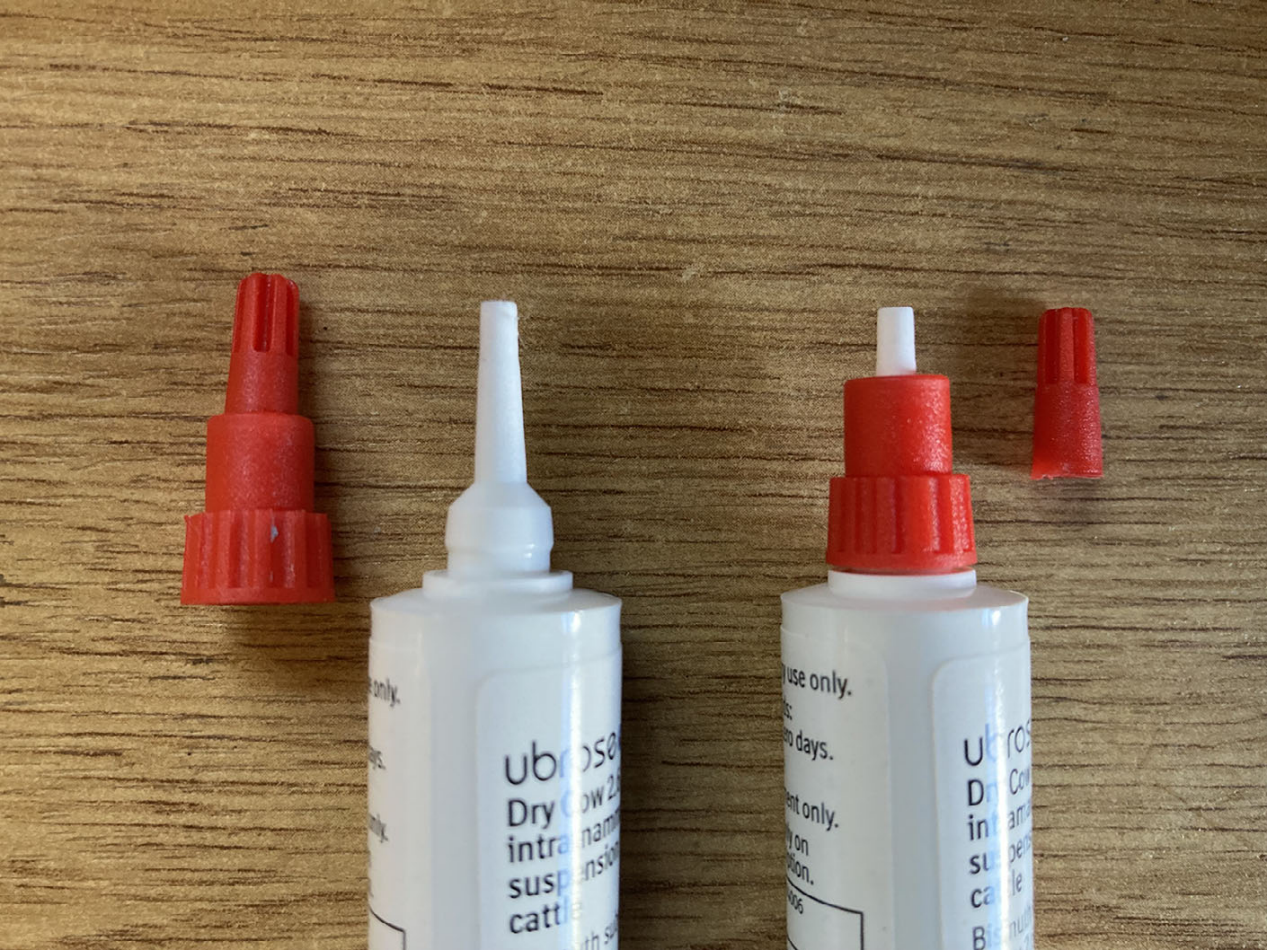
Teat sealant tubes used in the study, showing complete removal of cap for full insertion (left) and partial removal of cap for partial insertion (right)

A parallel randomised control trial design was used; cows were enrolled when they were selected by the farmer for drying off as per standard farm protocols. At each farm, the farmer identified which cows were to be dried off each week and dictated whether each cow would receive ITS only (Ubroseal Boehringer Ingelheim Animal Health UK) or intramammary AB and ITS, according to the protocols laid out in the farm's herd health plan. The cows were then randomised to receive ITS or AB + ITS via either the FI or PI of the cannulas. Randomisation was achieved by creating computer‐generated random lists of insertion types (FI or PI) for each farm and for each treatment type (ITS or AB + ITS). The next cow to be dried off was selected, and then the next line of the relevant list was exposed by the facilitator to show which insertion type it would receive; in this way, the facilitator was blinded to the insertion type until the cow was enrolled. Farm B opted to only allow enrolment of cows receiving ITS as the AB tubes used at drying off did not have the option of partial cannula insertion. FI involved removing the entire cap from the teat sealant tube (Figure 1, left) and inserting the full length of the cannula. PI was achieved by removing only the top section of the cap (Figure 1, right) and inserting only the exposed portion of cannula into the teat. The facilitator performed all of the drying off on all farms and was trained in best practice aseptic technique, as laid out by the AHDB for drying off cows, by three different experts in the field and was supervised initially for enrolment and drying off.^10^ On each farm, the drying off took place in the parlour at the end of morning milking.

All farms regularly completed milk records, and the SCC data collected were collated along with information on cases of clinical mastitis occurring within 30 days of calving. For the purposes of this study, clinical mastitis was defined as mastitis detected by farm staff (as per the herd health plan). The new infection rate (cows with SCC <200k cells/mL before drying off having a first test of SCC >200k cells/mL after calving) and cure rate (cows with SCC >200k cells/mL before drying off having a first test of SCC <200k cells/mL after calving) were calculated from these data. Data were also collected on calving season, lactation number and reason for culling.

The SCC data were (naturally) log transformed, checked for symmetrical distribution and tested for normality using Welch's *t*‐test. Multivariable regression analyses were employed for data analysis; four logistic regression models were built, with postcalving SCC, new infection rate, cure rate or clinical mastitis cases as the dependent variable. The independent variable of interest, insertion type, as well as a priori independent variables, namely, farm, calving season and lactation number were used to build the model. For the models of postcalving SCC and clinical mastitis cases, precalving SCC was included if statistically significant. Interaction terms with theoretical plausibility were tested and included if significant. *p*‐Values of less than 0.05 were considered statistically significant throughout. All statistical analyses were performed using R statistical software (v4.3.1)^11^ with the Tidyverse^12^ and stats^11^ packages.

The null hypothesis was that there is no difference in postcalving SCC, new infection rate, cure rate or mastitis cases when comparing FI with PI.

## RESULTS

There was no evidence to allow us to reject the null hypothesis; that is, there was no difference in postcalving SCC, new infection rate, cure rate or mastitis cases when comparing FI with PI.

A total of 287 cows were enrolled in the study, and complete data were collected for 273 cows. Fourteen cows with missing data were excluded from the analysis; no calving date was recorded for three cows (two FI and one PI), 10 cows calved but left the farm before their first SCC postcalving (three FI and seven PI) and one cow calved and remained on the farm but no SCC postcalving was recorded (one FI). For the 14 cows that were excluded after enrolment, udder health was not recorded as a reason for leaving the farm. Of the 273 cows that were included in the study, 47.3% received FI of the cannula/e (*n* = 129) and 28.2% received AB + ITS as allocated by the farmers (*n* = 77). Due to the seasonal calving pattern at one of the three farms, 54.6% of cows enrolled were at the end of their first lactation (*n* = 149). Eighty‐one of the cows were from farm A (29.7%), 141 from farm B (51.6%) and 51 from farm C (18.7%) (Table 1).

**TABLE 1:** Description of cows allocated to full insertion and partial insertion groups

	Full insertion (*n* = 129)	Partial insertion (*n* = 144)
Farm		
A	40 (31.0%)	41 (28.5%)
B	70 (54.3%)	71 (49.3%)
C	19 (14.7%)	32 (22.2%)
Drying off category		
ITS	86 (66.7%)	110 (76.4%)
AB + ITS	43 (33.3%)	34 (23.6%)
Calving season		
Spring/summer	76 (58.9%)	102 (70.8%)
Autumn	53 (41.1%)	42 (29.2%)
Lactation number		
First	67 (51.9%)	82 (56.9%)
Second or later	62 (48.1%)	62 (43.1%)
SCC before drying off		
Low	104 (80.6%)	126 (87.5%)
High	25 (19.4%)	18 (12.5%)
SCC postcalving		
Low	112 (86.8%)	125 (86.8%)
High	17 (13.2%)	19 (13.2%)
Clinical mastitis		
Clinical mastitis cases	6 (4.7%)	7 (4.9%)

Abbreviations: AB, antibiotic; ITS, internal teat sealant; SCC, somatic cell count.

Milk recording occurred on average (mean) 14.6 days before drying off (standard deviation [SD]: 10.4 days) and 27.8 days after calving (SD: 17.9 days). Two hundred and thirty cows had low SCC before drying off, of which 206 continued to have low SCC postcalving, and 43 cows had high SCC before drying off, of which 31 continued to have high SCC postcalving (Table 2).

**TABLE 2:** Somatic cell count (SCC) changes during the dry period

SCC before drying off	SCC postcalving	Number of cows (*n* = 273)
Low	Low	206 (75.5%)
Low	High	24 (8.8%)
High	Low	12 (4.4%)
High	High	31 (11.4%)

**FIGURE 2:**
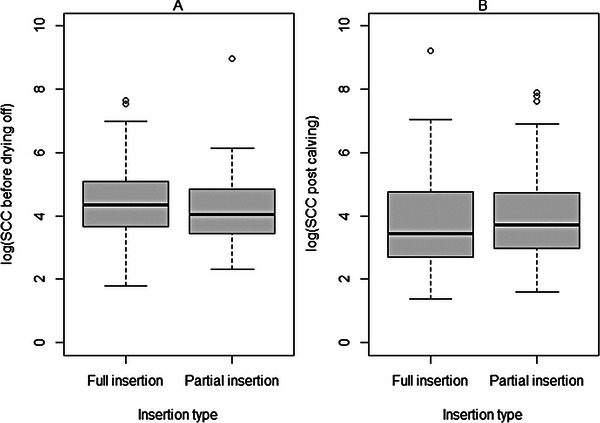
Boxplots showing somatic cell count (SCC) on a log10 scale (a) before drying off and (b) postcalving, by insertion type. The box shows the lower quartile (*Q*1), median (bold line) and upper quartile (*Q*3), and the whiskers show 1.5 the interquartile range (IQR) below or above *Q*1/*Q*3, respectively (i.e., *Q*1 ‒ 1.5 × IQR and *Q*3 + 1.5 × IQR). Outliers outside the whiskers are shown as data points

*t*‐Tests were performed to compare log SCC values across the two insertion groups. As expected, due to the randomisation protocol, the mean log SCC before drying off was not significantly different across the two groups: 4.41 for FI versus 4.16 for PI (SD: 1.18 and 0.98, respectively, *p* = 0.66). The mean log postcalving SCC was also not statistically significantly different across the two groups: 3.76 for FI versus 3.93 for PI (SD: 1.36 and 1.30, respectively, *p* = 0.29; Figure 2).

### Postcalving SCC

A total of 273 cows were included in a model of postcalving SCC, with 36 of the cows recorded as having high SCC postcalving. Variables of farm, calving season, infection status at drying off and lactation number were included; interaction terms were not significant. Factors associated with a high SCC postcalving were calving season, infection status before drying off and lactation number. Cows calving in spring/summer were more likely to have a high SCC postcalving than those who calved in the autumn (odds ratio [OR]: 4.61, 95% confidence interval [CI]: 1.77‒14.64, *p* = 0.004). Cows with a high SCC before drying off were 3.61 times more likely to have a high SCC postcalving (95% CI: 1.45‒8.94, *p* = 0.005). Cows in the second or later lactation were 3.08 times more likely to have a high SCC postcalving than cows in their first lactation (95% CI: 1.37‒7.31, *p* = 0.008). Farm was not significantly associated with postcalving SCC. Insertion type was also not statistically significantly associated with this outcome (OR: 1.13, 95% CI: 0.53‒2.44, *p* = 0.756; Table 3).

**TABLE 3:** Factors associated with high somatic cell count (SCC) postcalving

	Adjusted odds ratio (95% confidence interval)	*p*‐Value
Farm		
Farm A	Reference	
Farm B	0.31 (0.07‒1.07)	0.081
Farm C	0.91 (0.39‒2.19)	0.835
Calving season		
Autumn	Reference	
Spring/summer	4.61 (1.77‒14.64)	0.004
Infection status before drying off		
Low SCC at drying off	Reference	
High SCC at drying off	3.61 (1.45‒8.94)	0.005
Lactation number		
First lactation	Reference	
Second or later lactation	3.08 (1.37‒7.31)	0.008
Insertion type		
Full insertion	Reference	
Partial insertion	1.13 (0.53‒2.44)	0.756

### New infection rate

A total of 230 cows with low SCC before drying off were included in a model with new infection rate as the outcome variable; 24 cows were newly infected after calving (i.e., high SCC postcalving). Variables of farm, calving season and lactation number were included; interaction terms were not significant. Calving season was the only factor associated with new infection rate, with cows calving in the spring/summer more likely to be affected than cows calving in the autumn (OR: 3.70, 95% CI: 1.19‒16.27, *p* = 0.042). Cows in their second or later lactation were more likely to acquire a new infection than cows in their first lactation, although this difference was not statistically significant (OR: 2.45, 95% CI: 0.99‒6.28, *p* = 0.055). Farm was not associated with new infection rate. Insertion type was also not statistically significantly associated with this outcome (OR: 1.01, 95% CI: 0.42‒2.48, *p* = 0.989; Table 4).

**TABLE 4:** Factors associated with new infection rate in cows with a low somatic cell count (SCC) before calving

	Adjusted odds ratio (95% confidence interval)	*p*‐Value
Farm		
Farm A	Reference	
Farm B	0.24 (0.01–1.39)	0.184
Farm C	1.13 (0.44–3.04)	0.803
Calving season		
Autumn	Reference	
Spring/summer	3.70 (1.19–16.27)	0.042
Lactation number		
First lactation	Reference	
Second or later lactation	2.45 (0.99–6.28)	0.055
Insertion type		
Full insertion	Reference	
Partial insertion	1.01 (0.42–2.48)	0.989

### Cure rate

Forty‐three cows with high SCC before drying off were included in a model with cure rate as the outcome variable; 12 cows were cured after calving (i.e., low SCC postcalving). Variables of farm, calving season and lactation number were included; interaction terms were not significant. Cows calving in spring/summer were 7.63 times more likely to remain infected than those calving in autumn (95% CI: 1.29‒74.76, *p* = 0.042). Cows in their second or greater lactation were 9.34 times more likely to remain infected than cows in their first lactation (95% CI: 1.13‒212.14, *p* = 0.070), but this was not statistically significant. Farm was not associated with cure rate. Insertion type was also not significantly associated with the cure rate postcalving (OR: 1.46, 95% CI: 0.29‒7.37, *p* = 0.640; Table 5).

**TABLE 5:** Factors associated with cure rate in cows with a high somatic cell count (SCC) before calving

	Adjusted odds ratio (95% confidence interval)	*p*‐Value
Farm		
Farm A	Reference	
Farm B	0.18 (0.01–1.56)	0.140
Farm C	0.43 (0.04–3.43)	0.435
Calving season		
Autumn	Reference	
Spring/summer	7.63 (1.29–74.76)	0.042
Lactation number		
First lactation	Reference	
Second or later lactation	9.34 (1.13–212.14)	0.070
Insertion type		
Full insertion	Reference	
Partial insertion	1.46 (0.29–7.37)	0.640

### Clinical mastitis cases

A total of 273 cows were included in a model of clinical mastitis cases in the first month after calving; 13 cows had clinical mastitis in the first month after their calving. Variables of farm, calving season and lactation number were included; interaction terms were not significant. Lactation number was associated with clinical mastitis cases, with cows in their second or later lactation being 5.55 times more likely to be detected with clinical mastitis the first month after calving compared to cows in their first lactation (95% CI: 1.57‒26.12, *p* = 0.014). Cows that calved in spring/summer were less likely to be detected with clinical mastitis after calving than cows that calved in autumn, although this was not statistically significant (OR: 0.79, 95% CI: 0.24‒2.77, *p* = 0.692). Farm was not significantly associated with clinical mastitis cases. Insertion type was also not significantly associated with mastitis cases (OR: 1.24, 95% CI: 0.39‒4.11, *p* = 0.719; Table 6).

**TABLE 6:** Factors associated with clinical mastitis cases in the first month after calving

	Adjusted odds ratio (95% confidence interval)	*p*‐Value
Farm		
Farm A	Reference	
Farm B	1.08 (0.14–6.88)	0.936
Farm C	2.58 (0.68–12.57)	0.188
Calving season		
Autumn	Reference	
Spring/summer	0.79 (0.24–2.77)	0.692
Lactation number		
First lactation	Reference	
Second or later lactation	5.55 (1.57–26.12)	0.014
Insertion type		
Full insertion	Reference	
Partial insertion	1.24 (0.39–4.11)	0.719

## DISCUSSION

In this study, there was no difference in postcalving SCC, new infection rate, cure rate or clinical mastitis cases when comparing FI to PI. This differs from previous studies, which found that FI was associated with higher incidences of mastitis; however, these studies were using intramammary AB rather than ITS.^8^, ^9^ The difference between the results of our study and those of previous studies may be due to a number of factors; primarily, we looked at the effect of FI versus PI when using ITS either solely or in conjunction with intramammary AB as opposed to solely AB. Damage to the keratin lining has been cited as a potential cause of intramammary infections when using FI with intramammary AB; however, it is possible that when using ITS the importance of this lining is reduced. In addition, one of the studies involving comparing FI and PI was performed on Jersey cows,^9^ while the other was performed on majority crossbred Holstein–Friesian herds.^8^ Both of these breeds are smaller than pedigree Holsteins and are likely to have smaller teats, increasing the likelihood of damage from a tube cannula.^13^

Calving season was associated with high SCC postcalving and new infection rate, with cows calving in spring/summer more likely to have high SCC postcalving and new infections postcalving. Multiple studies have found an association between higher SCC and warmer months of the year.^14^, ^15^ However, the effect of calving season in our study was influenced by the largest farm in the study having an increase in calving numbers during the spring/summer, leading to more crowded conditions during the dry period.

Lactation number was associated with high SCC postcalving and clinical mastitis in the first 30 days postcalving, with cows in the second or later lactation more likely to have high SCC postcalving and clinical mastitis. In previous studies, lactation number has also been associated with higher SCC and increased rates of mastitis.^8^, ^16^, ^17^, ^18^

Infection status before drying off was associated with high SCC postcalving, with cows with high SCC before drying off being 2.87 times more likely to have high SCC postcalving. Infection status before drying off has also been associated with higher levels of mastitis postcalving in other studies.^16^, ^19^ Our study used the last milk recording before drying off as the indicator of infection status. Another approach would be to use any of the last three milk recordings with SCC above 200,000 cells/ml (possibly in conjunction with history of clinical mastitis in the last lactation), which would lead to higher sensitivity of infection status before drying off.

However, it should be noted that, due to time and recruitment constraints, our study failed to recruit 250 cows per group, leading to underpowered statistical analyses. This could could explain why no difference was detected between the two study groups. Future studies involving larger clinical trials, ideally with a range of breed and management systems, would be useful to further investigate the findings of our study.

In conclusion, this study showed that when a robust aseptic technique is used for drying off Holstein cows, there was no difference in postcalving SCC, new infection rate, cure rate or mastitis cases when comparing FI and PI. However, larger studies are needed to corroborate these findings.

## AUTHOR CONTRIBUTIONS

*Data curation, formal analysis, investigation, project administration, writing—original draft preparation and writing—review and editing*: Cherrill Bedford. *Conceptualisation, methodology and writing—review and editing*: Philippa Jane Mahen. *Conceptualisation, funding acquisition, resources and writing—review and editing*: Kath Aplin. *Conceptualisation, funding acquisition, methodology, supervision and writing—review and editing*: George Oikonomou.

## CONFLICT OF INTEREST STATEMENT

The authors declare they have no conflicts of interest.

## ETHICS STATEMENT

The study was approved by the University of Liverpool Veterinary Research Ethics Committee (reference VREC733).

## Data Availability

The data that support the findings of this study are available from the corresponding author upon reasonable request.
